# A Senescence-Associated Gene Signature for Prognostic Prediction and Therapeutic Targeting in Adrenocortical Carcinoma

**DOI:** 10.3390/biomedicines13040894

**Published:** 2025-04-08

**Authors:** Hangya Peng, Qiujing Chen, Lei Ye, Weiqing Wang

**Affiliations:** 1 Department of Endocrine and Metabolic Diseases, Shanghai Institute of Endocrine and Metabolic Diseases, Ruijin Hospital, Shanghai Jiao Tong University School of Medicine, Shanghai 200025, China; xipenpen2014@163.com (H.P.); cqj40575@rjh.com.cn (Q.C.); 2 Shanghai National Clinical Research Center for Metabolic Diseases, Key Laboratory for Endocrine and Metabolic Diseases of the National Health Commission of the PR China, Shanghai Key Laboratory for Endocrine Tumor, Ruijin Hospital, Shanghai Jiao Tong University School of Medicine, Shanghai 200025, China; 3 Institute of Cardiovascular Diseases, Shanghai Jiao Tong University School of Medicine, Shanghai 200025, China

**Keywords:** adrenocortical carcinoma, senescence, prognosis, immune microenvironment, drug sensitivity

## Abstract

**Background/Objectives**: Cellular senescence plays a critical role in tumorigenesis, immune cell infiltration, and treatment response. Adrenocortical carcinoma (ACC) is a malignant tumor that lacks effective therapies. This study aimed to construct and validate a senescence-related gene signature as an independent prognostic predictor for ACC and explore its impact on the tumor microenvironment, immunotherapy, and chemotherapy response. **Methods**: Data were collected from The Cancer Genome Atlas (TCGA) and the Gene Expression Omnibus (GEO) database. Using Kaplan–Meier survival analysis, LASSO penalized Cox regression and multivariable Cox regression, we identified a prognostic model with four senescence-related genes (HJURP, CDK1, FOXM1, and CHEK1). The model’s prognostic value was validated through survival analysis, risk score curves, and receiver operating characteristic (ROC) curves. Tumor mutation burden was assessed with maftools, and the tumor microenvironment was analyzed using CIBERSORT and ESTIMATE. Immune and chemotherapeutic responses were assessed through Tumor Immune Dysfunction and Exclusion (TIDE) and OncoPredict. **Results**: The risk score derived from our model showed a strong association with overall survival (OS) in ACC patients (*p* < 0.001, HR = 2.478). Higher risk scores were correlated with more advanced tumor stages and a greater frequency of somatic mutations. Differentially expressed genes (DEGs) that were downregulated in the high-risk group were significantly enriched in immune-related pathways. Furthermore, high-risk patients were predicted to have reduced sensitivity to immunotherapy (*p* = 0.02). Bioinformatics analysis identified potential chemotherapeutic agents, including BI-2536 and MIM1, as more effective treatment options for high-risk patients. **Conclusions**: Our findings indicate that this prognostic model may serve as a valuable tool for predicting overall survival (OS) and treatment responses in ACC patients, including those receiving chemotherapy and immunotherapy.

## 1. Introduction

Adrenocortical carcinoma (ACC) is a rare but aggressive endocrine cancer with limited treatment options. Its incidence is estimated at 0.7 to 2 cases per million annually, with a higher prevalence in females (female-to-male ratio of 1.5 to 2.5:1) and peak onset in the 40s and 50s [1,2,3]. While surgical resection remains the primary method of approach, recurrence rates are high, reaching up to 75% even after complete resection [2]. Currently, mitotane is the only FDA-approved drug specifically for ACC. For patients with low-grade, localized ACC, post-surgical surveillance is generally preferred over mitotane therapy [4], while effective options for advanced-stage ACC remain limited. Despite efforts to introduce new therapies for ACC—including combination chemotherapy (etoposide, doxorubicin, cisplatin, and mitotane) [5], immunotherapy [6,7], tyrosine kinase inhibitors [8,9], and radiotherapy [10,11]—none have demonstrated significant efficacy in improving outcomes. Therefore, enhanced prognostic tools and biomarkers to better stratify ACC patients are urgently needed to advance precision medicine in this challenging malignancy.

Cellular senescence, marked by an irreversible cessation of cell division, was initially regarded as a natural defense against tumor growth. However, recent studies have shown that senescent tumor cells can persist, releasing a collection of factors known as the senescence-associated secretory phenotype (SASP), which primarily comprises proinflammatory chemokines and cytokines [12,13,14]. These SASP factors are increasingly recognized as a double-edged sword in cancer, capable of both promoting anti-tumor immune cell infiltration and driving tumor progression by influencing the behavior of nearby cells [15,16]. As a result, some researchers suggest the following novel strategy: first induce tumor cell senescence with a senescence-inducing agent, followed by the targeted elimination of these senescent cells using senolytic modalities [17]. Recent animal studies on ACC have shown that genetic mutations can trigger cellular senescence, contributing to adrenal cancer development in older mice [18,19]. Further research has demonstrated an upregulation of the aging biomarkers p21 and p53 in ACC, along with elevated Ki-67 expression, when compared to adrenocortical adenomas (ACA) [20,21]. However, the exact role of senescence in ACC remains unclear, as it could function either as a protective or a risk factor. Therefore, we hypothesize that gene expression related to senescence may provide a foundation for developing a model to predict ACC progression and guide treatment strategies. In this study, we developed a four-gene signature model based on senescence-associated genes to predict outcomes in ACC. Using data from the TCGA-ACC and GEO databases, we assessed the correlation between these genes and ACC prognosis. Furthermore, we explored how the signature relates to clinical features, immune cell infiltration, immune therapy responses, and drug sensitivity in ACC patients. Our findings provide insights into the association between these genes and the prognosis of ACC, which may inform future studies on their potential role in cellular senescence and therapeutic strategies.

## 2. Materials and Methods

### 2.1. Data Acquisition

A cellular senescence-associated gene set, consisting of 278 genes (Supplementary Table S1), was sourced from the CellAge database (http://genomics.senescence.info/cells, accessed on 23 April 2023). To identify differentially expressed genes (DEGs) between ACC and normal adrenal gland tissue, RNA-seq data from the TCGA-ACC (n = 77) and Genotype-Tissue Expression (GTEx) (n = 126) datasets were obtained from the UCSC Toil RNA-seq Recompute project (https://xenabrowser.net/datapages/, accessed on 23 April 2023). Additionally, DEGs were derived from the GSE12368 and GSE143383 datasets. RNA sequencing data and patient clinical characteristics were collected from TCGA-ACC database to establish the construction cohort (https://cancergenome.nih.gov/, accessed on 23 April 2023). Transcriptomic microarray data and survival information for two validation cohorts were downloaded from the GEO database, specifically from the GSE10927 and GSE19750 datasets (https://www.ncbi.nlm.nih.gov/geo/, accessed on 23 April 2023).

### 2.2. Establishment and Validation of Aging-Associated Gene Signature

To identify DEGs between tumor and normal tissues, we conducted differential expression analysis on the TCGA-ACC and GTEx datasets using DESeq2, edgeR, and limma. The combined use of these three packages helped mitigate potential computational biases that could arise from relying on a single R package. Genes with an adjusted *p*-value < 0.05 and a log fold change (logFC) > 1 were selected as DEGs. To ensure the reliability of our DEG selection, we refined this list by intersecting DEGs from the three methods with those identified in the GSE12368 and GSE143383 datasets (analyzed with limma). This final intersected set of DEGs was visualized using a Venn diagram. Next, aging-associated prognostic genes were identified through univariate Cox regression analysis. We then intersected these prognostic genes with the final set of DEGs, followed by LASSO regression and Cox proportional hazards modeling (coxph) to construct our predictive model. The risk model was calculated as follows:

(1) $$\begin{array}{cc} risk\;score= & coefficient\;1\times Expression\;1\\ & \;\;\;+coefficient\;2\times Expression\;2+\ldots \\ & \;\;\;+coefficient\;i\times Expression\;i \end{array}$$

Multivariate Cox regression analyses were performed to assess the independence of the risk score in predicting survival outcomes. In both the construction (TCGA-ACC) and validation cohorts (GSE19750 and GSE10927), patients were divided into low-risk and high-risk groups based on the median risk score. Kaplan–Meier (KM) curves were generated using the survival package to assess survival outcomes for patients stratified by risk score. In addition, time-dependent receiver operating characteristic (ROC) curves were constructed with the survivalROC package to evaluate the predictive performance of the risk signature.

### 2.3. Mutation Analysis

The mutation waterfall plot illustrating the mutation profiles of TCGA-ACC patients across two risk subgroups was generated using the R package maftools.

### 2.4. Differential Gene and Enrichment Analysis Between High-Risk and Low-Risk Groups

Differential gene expression analysis between high-risk and low-risk groups was conducted using DESeq2, with a logFC threshold of 1 and a significance cutoff at *p* < 0.05. To investigate the biological functions of the identified DEGs, Gene Set Enrichment Analysis (GSEA) was performed to identify enriched pathways using the KEGG subset, while upregulated and downregulated genes in the high-risk group were analyzed through the WikiPathways subset. Additionally, Gene Ontology (GO), and the Kyoto Encyclopedia of Genes and Genomes (KEGG) were also performed with the clusterProfiler package.

### 2.5. Immune-Related Signature Profiling

We applied ssGSEA to analyze the gene expression signature of 28 distinct immune cell types (Supplementary Table S2), and used CIBERSORT (https://cibersort.stanford.edu, accessed on 23 April 2023) to assess immune cell infiltration based on the gene signatures of 22 immune cell types. Both methods were employed to comprehensively evaluate immune cell infiltration across different stratified risk groups. Additionally, ESTIMATE (Estimation of STromal and Immune cells in MAlignant Tumor tissues using Expression data) was used to evaluate tumor purity by quantifying the proportion of immune and stromal cells within the tumor microenvironment. We also systematically explored the correlations between senescence-related genes in our model and genes involved in antigen processing and presentation, immune checkpoint genes, and phagocytosis signaling.

### 2.6. Immunotherapy Response

Tumor Immune Dysfunction and Exclusion (TIDE) (http://tide.dfci.harvard.edu/, accessed on 23 April 2023) was employed to predict patient immunotherapy response by estimating multiple published transcriptomic biomarkers.

### 2.7. Drug Response Prediction

To identify potential drugs for high-risk patients, we employed the oncoPredict package (Version 1.2, updated on 5 April 2024) to predict the sensitivity of 198 drugs across two risk groups based on the transcriptomic data associated with half-maximal inhibitory concentration (IC50).

### 2.8. Statistical Analysis

**Figure 1:**
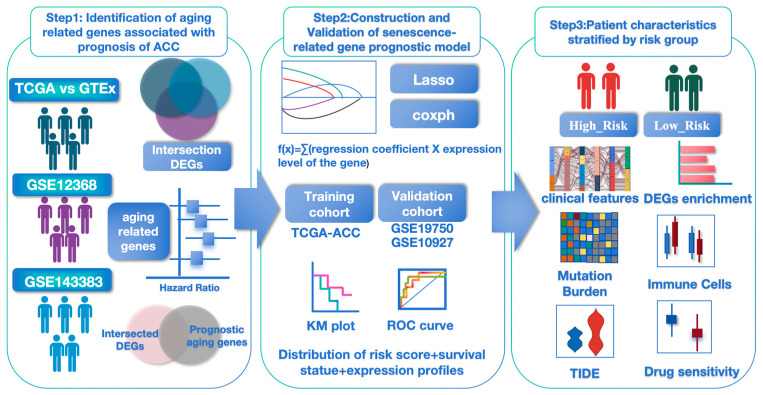
The flow of diagram of our study.

R version 4.4.0 and SPSS Statistics V27.0 were utilized for data analysis and graph generation. For comparing clinical characteristics between the high- and low-risk groups, an unpaired Student’s *t*-test was employed for normally distributed variables, while non-normally distributed variables were analyzed using the Wilcoxon rank-sum test. Categorical variables were compared using the χ^2^ test. Statistical significance was defined as *p* < 0.05. A flowchart of our study is shown in Figure 1.

## 3. Results

### 3.1. Aging-Related Prognostic Gene Screening and Senescence Model Construction in ACC

**Figure 2:**
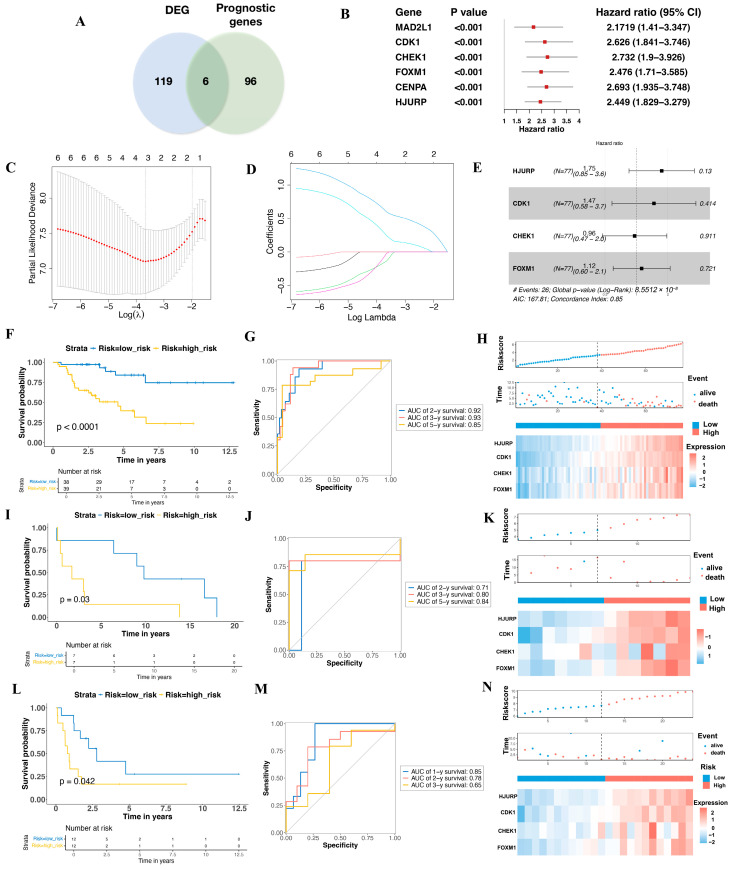
Construction and Validation of the Prognostic Model. (**A**) The Venn diagram highlights six differential expression genes associated with prognosis. (**B**) The forest plot displays the hazard ratios of the six genes in relation to overall survival (OS). (**C**) LASSO regression analysis of OS-related genes. (**D**) The cross-validation results for tuning parameter selection in the LASSO regression model. (**E**) The forest plot illustrating the multivariate Cox regression analysis of the four genes included in the final prognostic model. Kaplan–Meier survival curves, time-dependent ROC curves, distribution of risk scores, survival status, and expression profiles of the four signature genes for the TCGA-ACC dataset (**F**–**H**). Kaplan–Meier survival curves, time-dependent ROC curves, distribution of risk scores, survival status, and expression profiles of the four signature genes for the GSE19750 dataset (**I**–**K**). Kaplan–Meier survival curves, time-dependent ROC curves, distribution of risk scores, survival status, and expression profiles of the four signature genes for the GSE10927 (**L**–**N**).

Using RNA-seq count data from TCGA-ACC (n = 77) and GTEx (n = 126), we identified 2576 upregulated genes through an integrative analysis combining DESeq2, edgeR, and limma results (Supplementary Figure S1). We further refined these genes by incorporating upregulated genes identified from differential expression analysis of the GSE12368 (ACC vs. Normal, 12 vs. 6) (905 upregulated genes) (Supplementary Figure S2) and GSE143383 (ACC vs. Normal, 57 vs. 5) (333 upregulated genes) (Supplementary Figure S3) datasets, narrowing the list to 125 genes (Supplementary Figure S4 and Table S3) for in-depth analysis. To explore the association between cellular senescence-related genes and survival in TCGA-ACC (n = 77, two patients diagnosed with sarcomatoid features were excluded), we performed univariate Cox proportional hazards regression and identified 102 genes with *p* < 0.05 (Supplementary Table S4). The intersection of these 125 genes with the 102 significant survival-associated genes resulted in six aging-related prognostic genes for further investigation (Figure 2A). To develop a prognostic gene signature, we refined these six candidate genes through Lasso regression analysis using an optimized λ value, resulting in a risk score formula based on the expression levels of HJURP, CDK1, FOXM1 and CHEK1 (Figure 2B–E). The risk score was calculated as follows:

(2) $$\begin{array}{cc} risk\;score= & \left(0.5541\times expression\;of\;HJURP\right)\\ & \;\;\;\;+\left(0.2132\times expression\;of\;CDK1\right)\\ & \;\;\;\;+(0.1329\times expression\;of\;FOXM1)\\ & \;\;\;\;+\left(0.001\times expression\;of\;CHEK1\right) \end{array}$$

This formula enables patient stratification based on gene expression levels relative to the median risk score, providing valuable insights into potential prognostic outcomes. As demonstrated in the KM plot, patients with higher risk scores had significantly poorer survival rates than those with lower scores (*p* < 0.001) (Figure 2F). The ROC analysis revealed areas under the curve (AUC) of 0.92, 0.93, and 0.85 for 2-, 3-, and 5-year survival, respectively (Figure 2G). Moreover, the distribution of risk scores, survival status, and gene expression heatmap for TCGA-ACC patients further highlight the association between high-risk status and poorer prognosis (Figure 2H). The association between the expression of these four genes and survival status in ACC patients is detailed in Supplementary Figure S5.

### 3.2. Validation the Prognostic Value of the Aging-Related Gene Signature in Two Independent GEO Cohorts

Two datasets from the GEO database were included to evaluate the effectiveness of the risk model: GSE19750 (n = 14, platform: GPL570, raw data in Supplementary Table S5) and GSE10927 (n = 24, platform: GPL570, raw data in Supplementary Table S6). Consistent with the predictive cohort, both validation groups showed that patients in the higher-risk category had worse overall survival rates. Notably, the KM plot for GSE19750 displayed a significant difference between the two risk groups (*p* = 0.03) (Figure 2I), with ROC curve analysis being performed to assess the model’s predictive ability, yielding AUC values of 0.71, 0.80, and 0.84 for 2-, 3-, and 5-year survival in GSE19750, respectively (Figure 2J). The distribution maps of risk score and survival time for GSE19750 further illustrate the model’s capacity to differentiate patient survival status based on the expression of HJURP, CDK1, FOXM1, and CHEK1 (Figure 2K). Similar findings were observed in GSE10927, with the high-risk group showing worse overall survival (OS) compared to the low-risk group (*p* = 0.042) (Figure 2L). Due to the limited number of patients surviving beyond three years (mean survival: 2.49 ± 0.59 years), ROC curve analysis of GSE10927 was conducted for 1-, 2-, and 3-year survival, yielding AUC values of 0.85, 0.78, and 0.65, respectively (Figure 2M). The lower AUC for 3-year survival may be attributed to the small number of long-term survivors, with only five patients living beyond three years. Detailed survival data are provided in Supplementary Table S7. The survival outcome plots for GSE10927 further highlight how the model classifies patient survival based on the expression levels of HJURP, CDK1, FOXM1, and CHEK1, reinforcing the model’s predictive capacity (Figure 2N).

### 3.3. Multivariate Cox Proportional Hazards Regression Analysis of the Risk Score

**Figure 3:**
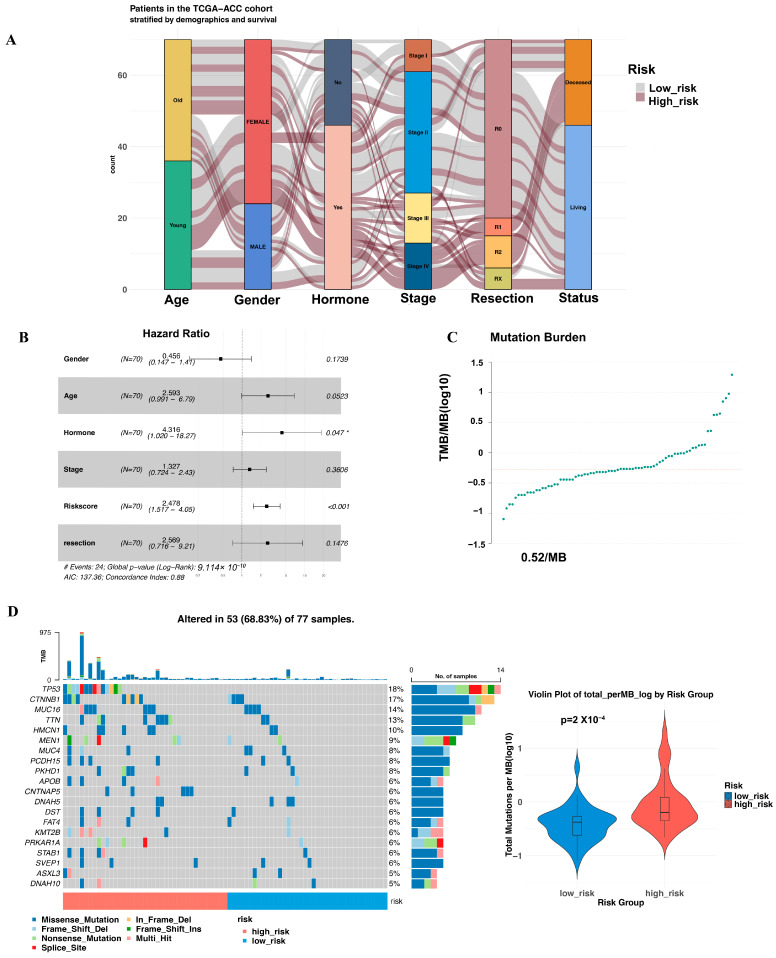
Clinical features and mutation profile of ACC patients in the two risk groups. (**A**) The Sankey diagram shows clinical traits across the two risk groups. (**B**) The forest plot depicts the results of a multivariate Cox regression analysis, highlighting the association between clinical characteristics and risk score with overall survival.* *p* < 0.05 (**C**) The mutation burden of TCGA-ACC dataset. (**D**) The waterfall plot visualizes the characteristics of the top 20 mutation types in the two groups with violin plot of mutation burden between the two cohorts.

A comparison of clinical characteristics—including age, gender, hormone secretion, tumor stage, and resection status—between the high- and low-risk groups (Table 1 and Figure 3A) revealed statistically significant differences in tumor stage and resection status (both *p* < 0.001). Multivariate Cox regression analysis confirmed that the risk score was an independent prognostic factor, unaffected by age, gender, hormone secretion, tumor stage, or resection status (Figure 3B). Due to missing information, the analysis was conducted on a subset of 70 patients (Supplementary Table S8). No statistically significant differences were observed in treatment regimens between the two groups (detailed in Supplementary Table S9).

### 3.4. Mutation Landscape Across the Two Groups

Among the 77 patients in the TCGA-ACC project, 68.83% (n = 53) exhibited somatic mutations, most of which were missense mutations, with an average mutation burden of 0.52/MB (Figure 3C,D). The three most frequently mutated genes were TP53 (18%), CTNNB1 (17%), and MUC16 (14%). Notably, high-risk patients showed a higher mutation burden compared to low-risk patients (Figure 3D).

### 3.5. Pathway Enrichment Analysis of DEGs Expression Between High- and Low-Risk Cohorts

**Figure 4:**
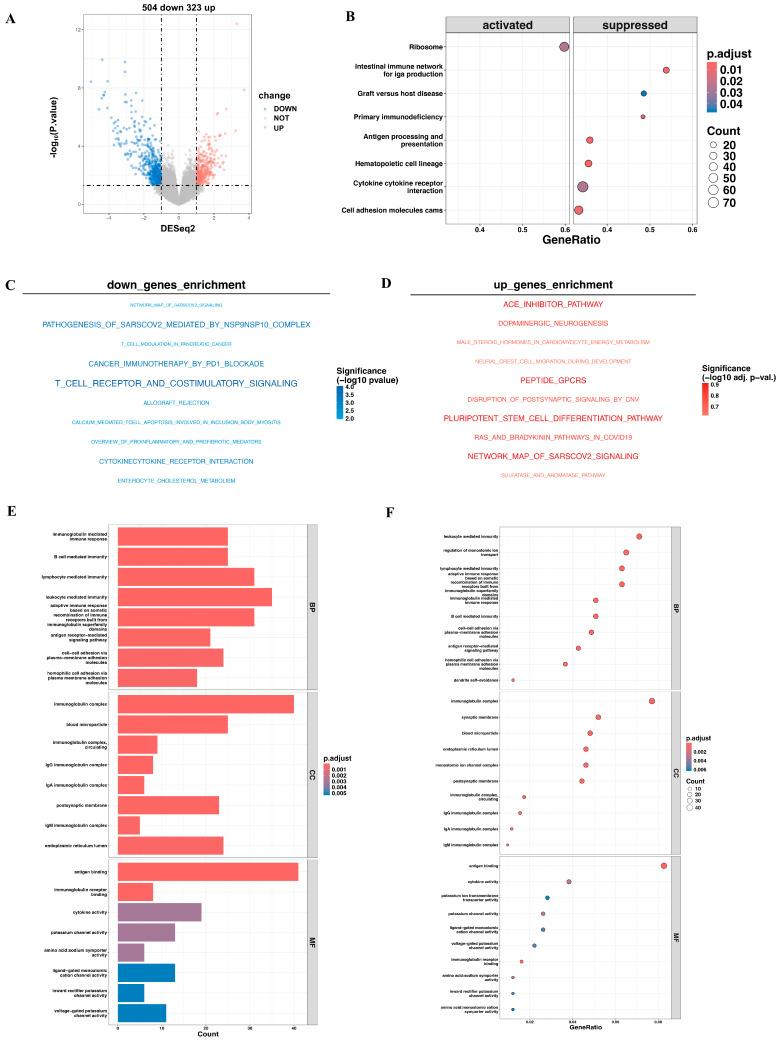
DEGs and related enrichment pathways between high-risk and low-risk groups. (**A**) The volcano plot illustrates the differentially expressed genes between the high-risk and low-risk groups. (**B**) The dot plot of Gene Set Enrichment Analysis (GSEA) shows the enriched pathways in the differentially expressed genes. (**C**,**D**) The enrichment analysis of the pathways associated with downregulated and upregulated genes in the high-risk group. (**E**,**F**) The GO enrichment analysis of the DEGs highlights biological processes, molecular functions, and cellular components.

To gain deeper insight into the mechanisms underlying outcome differences between these two risk categories, we applied the DESeq2 package, identifying 323 upregulated and 504 downregulated genes in high-risk patients compared to low-risk patients (Figure 4A). Notably, GSEA revealed the suppression of several pathways related to immune system function, including antigen processing and presentation, cytokine–cytokine receptor interaction and the intestinal immune network for IgA production when considering all DEGs (Figure 4B). Similarly, genes downregulated in the high-risk group were found to be enriched in immune-related pathways, particularly T cell receptor and costimulatory signaling, as well as cancer immunotherapy through PD-1 blockade, both of which are closely associated with tumor immunotherapy (Figure 4C). Conversely, while upregulated genes in the high-risk group showed enrichment in certain pathways, these pathways did not reach statistical significance after adjustment for multiple comparisons (Figure 4D). GO analyses of all DEGs identified immune-related pathways, with GO terms such as “antigen receptor-mediated signaling pathway”, “lymphocyte mediated immunity” and “antigen binding” (Figure 4E,F) as well as KEGG analyses as “cytokine-cytokine receptor interaction” when analyzing all DEGs (Supplementary Figure S6).

### 3.6. Tumor-Infiltrating Immune Cell Profile

**Figure 5:**
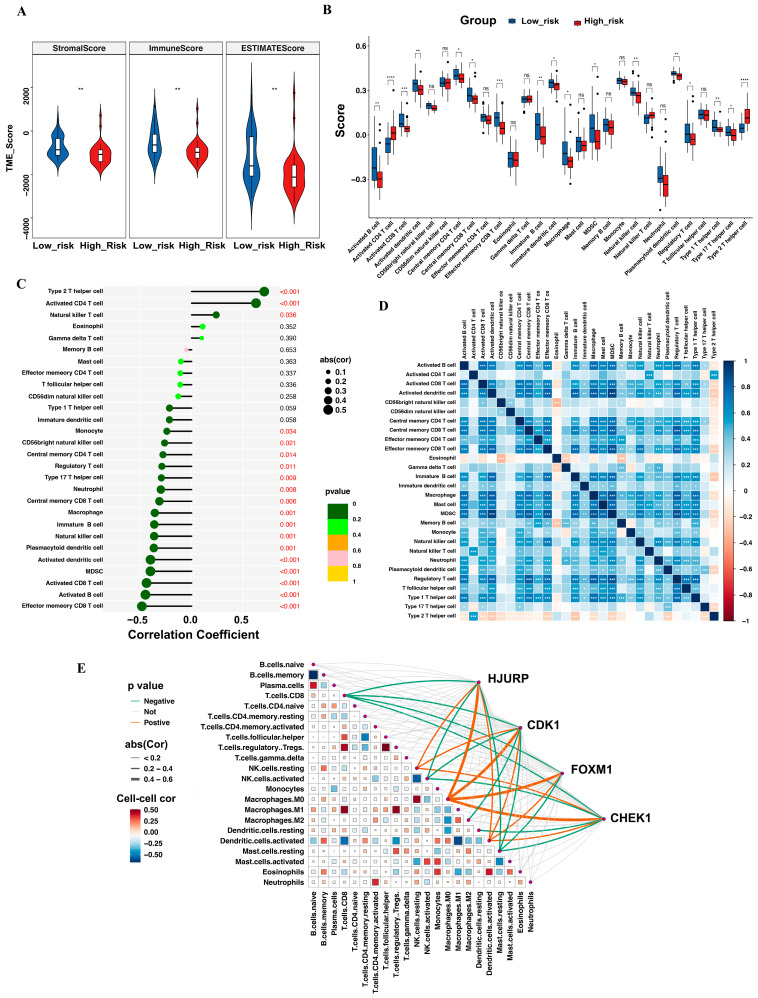
Comparison of tumor microenvironment features between the two risk cohorts. (**A**) The violin plot illustrates the stromal score, immune score, and ESTIMATE score between the high- and low-risk groups. (**B**) The box plot shows the scores of 28 immune cell types in the two risk groups. (**C**) The correlation analysis between the risk score and the 28 immune cell types (statistically significant results in red). (**D**) The heatmap visualization of the correlation among immune cells in ACC. (**E**) The heatmap demonstrates the relationship between the proportions of 22 immune cells in the TME and the genes from our model. * *p* < 0.05, ** *p* < 0.01, *** *p* < 0.001, **** *p*< 0.0001, ns: no statistical significance.

ESTIMATE analysis revealed a lower immune score in the high-risk group, suggesting lower immune cell infiltration in the high-risk group (Figure 5A). Consistently, ssGSEA confirmed significantly lower scores for activated CD8 T cells, effector memory CD8 T cells, and γδ T cells in the high-risk group. In contrast, type 2 T helper cells and activated CD4 T cells were more abundant in the high-risk group (Figure 5B). In line with these findings, a detailed correlation analysis between the risk score and immune cells is presented in Figure 5C, showing that most immune cell populations exhibited negative correlations with the risk score. The 28 tumor-infiltrating immune cell types displayed weak to moderate correlations (Figure 5D). Moreover, immune cell proportion composition analysis using CIBERSORT showed CD8 T cells, activated NK cells, and resting mast cells were all negatively correlated with genes in our model (HJURP, CDK1, FOXM1, CHEK1), while macrophages M0, activated dendritic cells, resting NK cells and activated dentritic cells were positively correlated (Figure 5E). The correlations between these four genes and immune cell scores, as analyzed by ssGSEA, are provided in Supplementary Figure S7.

### 3.7. Evaluation of Immune Function and Prediction of Immunotherapy Response

**Figure 6:**
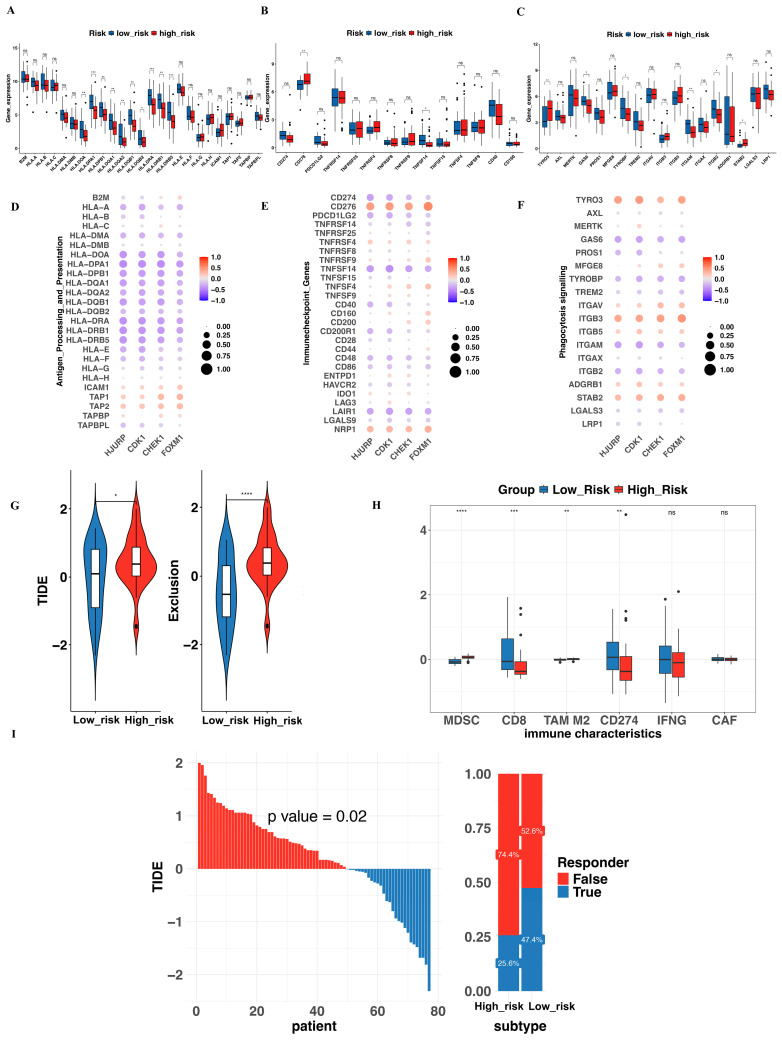
Analysis of immune function-related genes and model-based immunotherapy response prediction. (**A**) The box plot illustrates the expression of genes involved in antigen processing and presentation between the two risk groups. (**B**) Gene expression of immune checkpoint genes in the two risk cohorts. (**C**) Expression of phagocytosis signaling-related genes across high- and low-risk patients. (**D**–**F**) The correlation of HJURP, CDK1, FOXM1, and CHEK1 with genes involved in antigen processing and presentation, immune checkpoint regulation, and phagocytosis signaling. (**G**) The TIDE scores and immune exclusion scores between the two groups. (**H**) The immune characteristics of the tumor microenvironment (TME) in the two groups. (**I**) The immunotherapy response prediction for the two groups. * *p* < 0.05, ** *p* < 0.01, *** *p* < 0.001, **** *p* < 0.0001, ns: no statistical significance.

Tumor immune evasion operates through camouflage (avoiding recognition as malignant), coercion (preventing immune cell activation), and cryoprotection (shielding malignant cells from immune cytotoxicity) [22]. These mechanisms are largely governed by proteins expressed on the surfaces of both immune and tumor cells, such as PD-L1 and Human Leukocyte Antigen (HLA). To further explore the relationship between the risk score and immune function, we analyzed the correlation of mRNA expression of genes involved in antigen processing and presentation, immune checkpoints, and phagocytosis signaling with both the risk score and genes in our model. As shown in Figure 6A–F, most HLA genes (HLA-DOA, HLA-DPA1, HLA-DPB1, HLA-DQA1, HLA-DRA, HLA-DPB1, et al.) exhibited a negative correlation with the model genes. In contrast, immune checkpoints such as CD276 and NRP1 showed a strong positive relationship, while TNFSF14 and LAIR1 demonstrated a notable negative correlation. Among genes involved in phagocytosis signaling, ITGB3 and TYRO3 were positively correlated, while GAS6 and ITGAM were negatively associated. Detailed data are presented in Supplementary Table S10.

A higher TIDE score, accompanied by an elevated exclusion score, indicates reduced sensitivity to immunotherapy in the high-risk group (Figure 6G). Consistently, characteristics associated with Myeloid-Derived Suppressor Cells (MDSCs) and Tumor-Associated Macrophages M2 (TAM M2), both of which are known to play a crucial role in tumor immune evasion [23,24], were significantly elevated in the high-risk group based on our analysis (Figure 6H). Regarding the prediction of immunotherapy efficacy, 22.5% of high-risk patients respond to immunotherapy, compared to 46.2% in the low-risk group (*p* = 0.02) (Figure 6I). Data analyzed from TIDE was shown in Supplementary Figure S8 and Table S11.

### 3.8. Predictive Analysis for Drug Therapy

**Figure 7:**
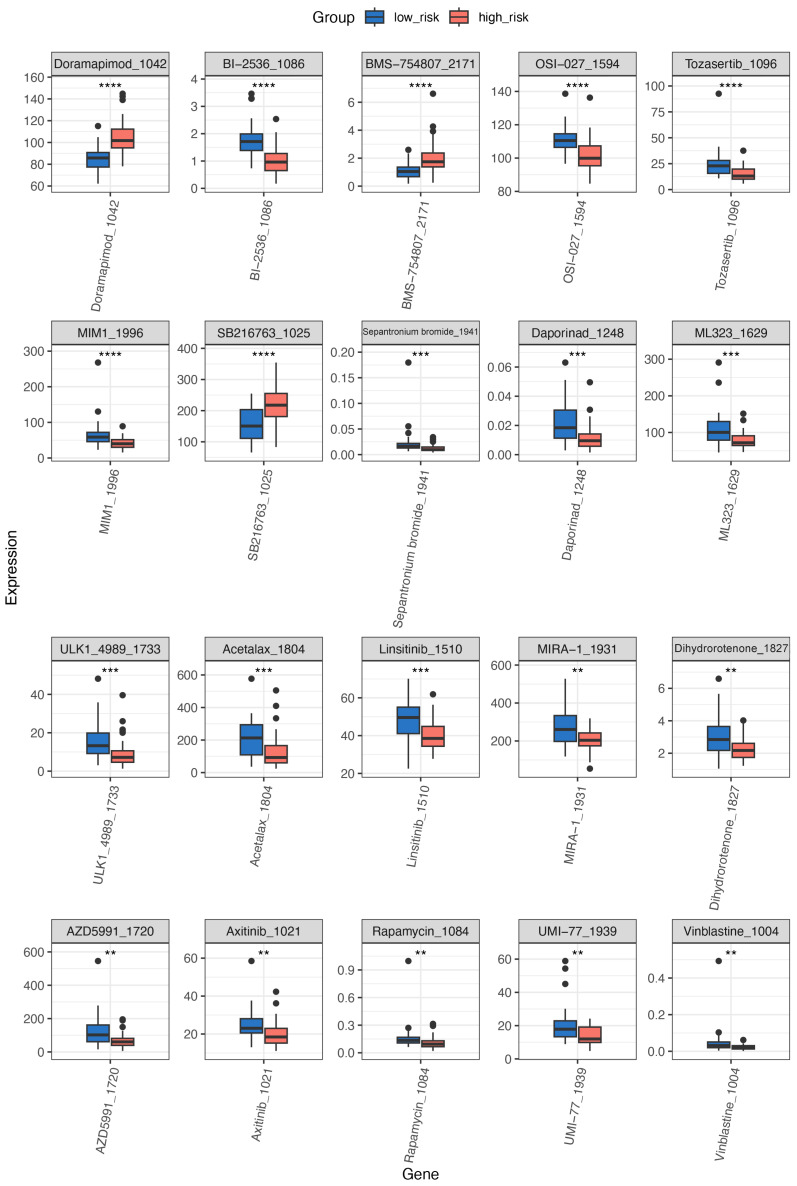
Prediction of drug sensitivity by OncoPredict in high-risk and low-risk groups. ** *p* < 0.01, *** *p* < 0.001, **** *p* < 0.0001. •: outliers.

OncoPredict predicts drug sensitivity based on gene expression levels. Out of 198 chemotherapeutic agents analyzed, 75 showed a significant difference in the predicted IC50 values between the two groups (*p* < 0.05). We highlight the top 20 drugs with the lowest *p*-values (*p* < 0.001) in Figure 7. The higher-risk group exhibited lower predicted IC50 values for BI-2536, OSI-027, Sepantronium bromide, MIM1, Tozasertib, Daporinad, ML323, Acetalax, Linsitinib, and ULK_4989 and others, compared to the lower-risk group, suggesting greater drug sensitivity compared to the lower-risk group. Detailed drug sensitivity data are provided in Supplementary Table S12, and additional results are shown in Supplementary Figure S9.

## 4. Discussion

As a malignant tumor originating from the adrenal cortex, the molecular landscape of ACC has been increasingly defined through multi-omics studies, facilitated by advances in detection technologies. Unfortunately, effective treatments for this rare and aggressive disease remain to be developed. Thus, there is an urgent need to discover reliable and complementary prognostic indicators and risk stratification approaches. The first report of cellular senescence dates back to the 1960s when scientists observed cell cycle arrest in human diploid fibroblasts [25]. Since then, the mechanisms underlying senescence in various biological processes have gradually been uncovered. Studies on senescence in adrenal diseases have shown that telomerase activity can serve as an effective tool for distinguishing malignant from benign adrenocortical tumors. Specifically, telomerase activity is higher in ACC than in adrenocortical adenomas (ACA) [26], making it a valuable marker for tumor classification. However, the exact role of senescence in ACC remains to be fully elucidated. In many other cancers, senescence has been reported to play a dual role [27]. On one hand, senescence contributes to tumorigenesis, as evidenced by its role in the invasion and progression of melanoma and papillary thyroid carcinoma (PTC) [28,29]. On the other hand, it acts as a tumor-suppressive mechanism by inducing cell-cycle arrest, thereby inhibiting tumor growth [30,31]. In our study, we utilized public databases (TCGA-ACC and GEO) to develop a model based on a four-gene signature associated with senescence (HJURP, CDK1, FOXM1, and CHEK1). Patients with a higher risk score calculated by our model exhibited significantly worse overall survival and more advanced tumor stages. Additionally, our analysis suggested a potential trend toward a reduced immunotherapy response in the high-risk group.

Each of the four genes (HJURP, CDK1, FOXM1, and CHEK1) identified in the model has been implicated in cell cycle regulation and tumor development. The Holliday junction recognition protein (HJURP) is a critical mitogenic protein that plays a vital role in nucleosome assembly during mitosis [32]. Chromosomal instability, a hallmark of cancer, results from persistent errors in chromosome segregation during cell division. HJURP is involved in the recruitment and assembly of the centromere and kinetochore, which are essential for accurate chromosome segregation, thereby playing a key role in maintaining chromosomal stability in tumor cells [33]. The upregulation of HJURP has been observed in various cancers, in which it regulates tumor progression and chemoresistance through multiple mechanisms, including the YAP1/NDRG1 transcriptional axis in triple-negative breast cancer [34], modulation of the MDM2/p53 pathway in pancreatic cancer [35], and promotion of CDKN1A degradation via the GSK3β/JNK pathway in prostate cancer cells [36]. Moreover, HJURP has been linked to poor prognosis in various cancers, including non-small cell lung cancer, hepatocellular carcinoma, and renal cell carcinoma [32,37,38]. Another gene in our model, cyclin-dependent kinase 1 (CDK1), is a key member of the cyclin-dependent kinase family and plays a pivotal role in regulating cell cycle progression. Specifically, its interaction with cyclin B, which peaks during the G2/M phase, ensures the orderly transition of cells into mitosis [39]. The overexpression of CDK1 has been observed in ACC cell lines, and where it has been shown to lock ACC cells at the G2/M checkpoint through interaction with UBE2C and AURKA/B. Additionally, CDK1 promotes epithelial-to-mesenchymal transition via Slug and Twist [40]. Recently, several CDK1 inhibitors have been developed as therapeutic strategies for cancer treatment. For instance, BEY1107, an orally active CDK1 inhibitor, has been evaluated in patients with locally advanced or metastatic pancreatic cancer (NCT03579836) [39]. Furthermore, forkhead Box M1 (FOXM1) is a transcription factor (TF) belonging to the forkhead box protein family (FOX). It plays a critical role in upregulating the expression of various genes associated with DNA replication, G1/S and G2/M transition. These include cyclin A2, Cdc25A phosphatase, and ATF2 [41,42]. Finally, CHEK1 serves as a crucial regulator of the DNA damage response (DDR), playing a vital role in detecting DNA replication stress caused by oncogene activation or dysfunction of the G1 checkpoint [43]. The CHK1/2 inhibitor AZD7762 has been shown to enhance anticancer efficacy synergistically when combined with other drugs [44]. Senescence occurs when cells respond to various stressors, such as DNA damage, triggering a cascade of signaling pathways which lead to sustained cell cycle arrest. This process results in a decline in cell cycle activity and the downregulation of mitotic proteins [45]. Therefore, we suggest the upregulation of genes in our model work together in regulating cell cycle and tumor progression, with HJURP ensuring chromosome stability, CDK1 driving mitotic entry, and FOXM1 promoting proliferation and CHEK1 supporting cell survival under DNA damage conditions.

It is well established that “cold” tumors, such as ACC, are characterized by low immune cell infiltration, which is associated with poorer outcomes and a diminished response to immunotherapy [46,47]. Clinical trials investigating immunotherapy in ACC have consistently shown limited efficacy, with low overall response rates and limited progression-free survival [48,49,50]. This resistance may be attributed to factors such as cortisol secretion [51] or specific mutation types. Notably, mutations in the Wnt/β-catenin pathway, one of the most common genetic alterations in ACC [52] (17% in our study), have been shown to impair anticancer immune responses [53,54,55]. Individuals in the high-risk group carry a greater burden of mutation types, and the enrichment of downregulated genes in this group suggests a suppressed immune function, particularly in pathways related to T cell activity, immunotherapy response, and cytokine signaling. These findings suggest that the poorer OS and more advanced tumor stage in the high-risk group may be driven by an abnormal immune cell distribution and functional impairments. This is further supported by reduced tumor purity, as calculated by ESTIMATE, and diminished immune cell infiltration, as revealed by ssGSEA and CIBERSORT, in the high-risk group. Among the immune cells, a notable reduction in activated CD8 T cells-the key effectors in tumor cell elimination partly explains the immune evasion observed in these patients. Additionally, the high-risk cohort exhibited decreased levels of activated dendritic cells, suggesting a deficiency in immune surveillance. Senescent tumor cells secrete chemokines that attract immune cells, particularly macrophages, to facilitate tumor cell elimination [56]. Consistent with our findings, high-risk patients exhibited fewer immune cell signatures in their transcriptomes, and the risk score was negatively correlated with macrophage signatures. This reduced immune infiltration may drive tumor cells in the high-risk group to favor cell cycle re-entry over senescence, potentially contributing to their increased proliferative capacity.

To further assess the immune response in the tumor microenvironment in relation to our model, we investigated genes involved in the recognition and elimination of tumor cells by immune cells. Human Leukocyte Antigen (HLA), which encodes the Major Histocompatibility Complex (MHC) proteins responsible for immune recognition, is inversely correlated with the genes from our model. Specifically, MHC class I molecules, such as HLA-A, interact with CD8+ T cells to facilitate tumor cell destruction, while MHC class II molecules—HLA-DPA1, HLA-DPB1, HLA-DQA1, HLA-DQB1, and HLA-DRB1—are predominantly expressed on antigen-presenting cells, such as dendritic cells and macrophages, where they are recognized by CD4+ T cells [57]. Regarding immune checkpoint genes, CD276 and NRP1 are positively correlated with the genes in our model, while TNFSF14 shows a negative correlation with these genes in the tumor immune microenvironment of high-risk patients. CD276 is reported to be expressed on the surface of cancer stem cells and tumor-associated macrophages, playing a critical role in suppressing the immune response against tumors [58,59]. NRP1 (Neuropilin-1) functions as an “immune memory checkpoint” limiting the development of long-lived tumor-specific T cells, which are essential for maintaining durable antitumor immunity [60]. Additionally, NRP1 promotes tumor cell survival and progression by interacting with the hypoxia response and microRNAs [61]. Alternatively, TNFSF14 (Tumor Necrosis Factor Superfamily Member 14), also known as LIGHT, enhances anti-tumor immune responses by promoting vascular normalization and the generation of tertiary lymphoid structures [62]. In terms of phagocytosis signaling, TYRO3 and ITGB3 are significantly associated with our model genes. TYRO3, a member of the TYRO3, AXL, and MerTK (TAM) receptor tyrosine kinase (RTK) family, is upregulated in various cancers [63]. It inhibits ferroptosis and suppresses innate immunity in TME, making it a potential predictive biomarker for identifying patients who may benefit from anti-PD-1/PD-L1 therapy [64]. ITGB3, an integrin that binds to fibronectin, vitronectin, and collagen, facilitates interactions between the cell cytoskeleton and the extracellular matrix [65]. A recent study has highlighted its pivotal role in mediating intracellular communication through extracellular vesicles, which are essential for cancer metastasis [66]. In summary, ACC tumors in high-risk patients tend to exhibit immune dysfunction through various mechanisms, potentially leading to greater resistance to immunotherapy compared to those with lower-risk groups.

As discussed above, high-risk patients with higher TIDE scores may exhibit a poorer response to immunotherapy, accompanied by an increased presence of MDSCs and TAM M2, both of which are immunosuppressive myeloid cells. MDSCs are primarily generated in the bone marrow, and upon migrating to the tumor site, they can differentiate into immunosuppressive macrophages through the activation of the transcription factor NF-κB by S100A8/A9 proteins [67]. Furthermore, the immunosuppressive activity of MDSCs can be induced by IFNγ mediated via the activation of the transcription factor STAT1 [68]. TAM M2 can activate Smad2/3 and Smad1/5/8, thereby promoting the epithelial–mesenchymal transition (EMT) and tumor metastasis [69]. Moreover, cytokines secreted by TAM M2, including TNF-α, ICAM-1, and IL-6, further contribute to tumor growth and invasion [70]. Recent studies have shown that targeting either MDSCs or M2 TAMs can enhance the efficacy of immunotherapy [71,72]. Based on our model’s high-risk score, machine learning analysis indicates that the upregulation of MDSCs and TAM M2, coupled with the downregulation of CD8+ T cells in the tumor microenvironment, may contribute to the diminished immunotherapy response observed in high-risk patients.

OncoPredict integrates data from the Cancer Cell Line Encyclopedia (CCLE) and the Genomics of Drug Sensitivity in Cancer (GDSC) to identify potent chemotherapy options. As indicated by the results, 10 out of the 20 drugs with a *p*-value < 0.001 demonstrated efficacy in the higher-risk group, exhibiting lower IC50 values. These drugs target key molecular pathways related to tumor cell proliferation, survival, and metabolism. Specifically, the cell cycle regulators are as follows: BI-2536 (targeting polo-like kinase 1) [73] and OSI-027 (an mTOR kinase inhibitor) [74]; the survival signaling pathways are targeted by Sepantronium bromide (targeting surviving) [75] and Tozasertib (targeting Aurora kinase B) [76]; Daporinad (inhibit NAD+ synthesis pathway) [77] affects metabolic pathways; and Linstinib (an oral kinase inhibitor for IGF-1R and the insulin receptor) [78] targets insulin/IGF signaling. Notably, all of these drugs have been registered for clinical trials. In contrast, MIM1 (Mcl-1 protein inhibitor) [79], ML323 (ubiquitin-specific protease 1 inhibitor) [80], Acetalax (a phenylpyridine compound) [81], and ULK1_4989 (targeting autophagy) [82] remain in preclinical development. We hypothesize that the model genes in the high-risk group primarily drive robust mitotic activity in tumor cells, a process that is strongly dependent on cell cycle and metabolic pathways. This may explain the increased sensitivity of the high-risk group to the predicted drugs. Among these, BI-2536 has been shown to exert cytotoxic effects on adrenocortical carcinoma cell lines (H295R and SW13) in both in vitro and in vivo studies, including cell line-derived xenograft (CDX) models in nude mice [83]. Additionally, it has been reported to induce senescence in colorectal cancer cell lines [84] suggesting that high-risk patients may be more susceptible to senescence induction.

There are several limitations in our study. First, the model was developed and validated using publicly available datasets, which are constrained by a relatively small sample size and a retrospective design, with some patients lacking comprehensive clinical data. Second, although TCGA data were used for model development and two independent GEO datasets for external validation, potential batch effects may arise due to variations in data generation platforms, processing methods, or technologies. Third, all our data were based on machine learning without experimental validation. To address these limitations, further validation in independent, larger cohorts with standardized protocols is essential to assess the model’s robustness and generalizability.

## 5. Conclusions

In summary, we have developed a four-gene signature related to the cell cycle that provides insights into the prognosis of adrenocortical carcinoma (ACC). Using this model, we also investigated clinical characteristics, transcriptomic features, immune profiles, and drug sensitivity predictions. Our analysis suggests that low-risk individuals, as stratified by our model, may demonstrate enhanced sensitivity to immunotherapy, while high-risk individuals may benefit more from alternative therapies, such as chemotherapy. This study represents the first attempt to categorize ACC based on the expression of senescence-related genes using bioinformatics tools, potentially opening new therapeutic avenues for ACC.

## Figures and Tables

**Table 1:** Clinical characteristics of high- and low-risk ACC patients.

	Low Risk Group (n = 36)	High Risk Group (n = 34)	*p* Value
Age	46.94 ± 2.62	46.84 ± 2.69	0.335
Gender			
Female	23 (63.89%)	23 (67.65%)	0.741
Male	13 (36.11%)	11 (32.35%)	
Hormone Secretion			
Yes	21 (58.33%)	25 (73.53%)	0.181
No	15 (41.67%)	9 (26.47%)	
ENSAT stage			<0.001
I	6 (16.67%)	3 (8.82%)	
II	24 (66.67%)	10 (29.41%)	
III	4 (11.10%)	10 (29.41%)	
IV	2 (5.56%)	11 (32.35%)	
Resection Status			<0.001
R0	34 (94.44%)	16 (47.05%)	
R1	0	5 (14.71%)	
R2	1 (2.78%)	8 (23.53%)	
Rx	1 (2.78%)	5 (14.71%)	

## Data Availability

The data and results utilized in this study were obtained from the following databases: TCGA (https://xenabrowser.net/datapages/, accessed on 23 April 2023), GTEx (https://xenabrowser.net/datapages/, accessed on 23 April 2023) and GEO (http://www.ncbi.nlm.nih.gov/geo/, accessed on 23 April 2023).

## Supplementary Materials

The following supporting information can be downloaded at: https://www.mdpi.com/article/10.3390/biomedicines13040894/s1, Figure S1: DEGs Analysis of TCGA-ACC and GTEx Datasets; Figure S2: DEGs analysis of the data derived from GSE12368; Figure S3: DEGs analysis of the data derived from GSE143383; Figure S4: Venn Diagrams of Upregulated Genes Across Different Analytical Methods and Databases; Figure S5: Kaplan-Meier (KM) plots depicting the relationship between HJURP, CDK1, FOXM1 and CHEK1 expression and survival in ACC patients; Figure S6: KEGG pathway enrichment analysis of the DEGs; Figure S7: Correlation analysis of 28 immune cell types with genes in our model; Figure S8: Waterfall plot of immunotherapy response in ACC patients stratified by our model, analyzed using TIDE; Figure S9: Boxplot of chemotherapy sensitivity (*p*-value < 0.05) in the two risk groups. Drugs ranked from 21 to 40 are listed. Table S1: A cellular senescence-associated gene set; Table S2: Gene signature of 28 distinct immune cells; Table S3: Gene list of 125 genes intersected by TCGA-GTEx, GSE12368 and GSE143383; Table S4: A list of 102 aging-related genes with prognostic significance; Table S5: Raw data of GSE19750; Table S6: Raw data of GSE10927; Table S7: The survival status, survival time, and risk information in patients from the three cohorts (TCGA-ACC, GSE19750, GSE10927); Table S8: Clinical characteristics of patients in TCGA-ACC; Table S9: Comparison of treatment regimens between the two groups; Table S10: The Correlation of genes from our model and genes invovled in antigen process and presentation, immuncheck ponits and phagocytosis signaling; Table S11: TIDE analysis data of 77 ACC patients; Table S12: *p*-values of 198 chemotherapeutic agents compared between the two risk groups.
